# A clinical picture of pulmonary embolism revealing light‐chain myeloma

**DOI:** 10.1002/ccr3.1234

**Published:** 2017-11-02

**Authors:** Badia Belarj, Amine El Alaoui, Souhail Dahraoui, Jean Uwingabiye, Emmanuel Millbank Owusu, Anas Rochdi, Asmaa Biaz, Abdellah Dami, Sanae Bouhsain, Zohra Ouzzif, Nawfel Doghmi, Samira El Machtani Idrissi

**Affiliations:** ^1^ Department of Clinical Biochemistry and Toxicology Faculty of Medicine and Pharmacy Mohammed V Military Teaching Hospital Mohammed V University Rabat Morocco; ^2^ Department of Intensive Care Units Faculty of Medicine and Pharmacy Mohammed V Military Teaching Hospital Mohammed V University Rabat Morocco

**Keywords:** Monoclonal‐free kappa light chain, multiple myeloma, pulmonary embolism

## Abstract

We are highlighting on the particularity of a clinical picture of pulmonary embolism revealing light‐chain myeloma in a 56‐year‐old male patient. Myeloma remains a rare affection. Even though its revelation through pulmonary embolism remains rare, it can be explained by hyperviscosity syndrome accompanying it.

## Introduction

Light‐chain multiple myeloma is a malignant hemopathy characterized by medullary invasion by tumor plasma cells, with the secretion of free light chains of immunoglobulins in the blood giving signs of bone disorders. However, it can be asymptomatic or can start by less clear clinical manifestations such as recurrent infections, anemic syndromes, renal failure, neurological symptoms, pathological fractures, or even amylosis 1. Revelation through pulmonary embolism is exceptional. Through this observation, we are emphasizing on the particularities of light‐chain myeloma.

## Case Report

A 56‐year‐old patient, who underwent an osteosynthesis operation 22 years ago, cholecystectomy a month earlier, and being treated on anticoagulants, admitted at the intensive care unit of Mohammed V Military Hospital for a sudden acute respiratory distress associated with thoracic pains, orthopnoea, and signs of confusion. Clinical examination following his admission showed a polypneic patient with crackling and snoring groans at the thoracic base. Blood test revealed a normochromic normocytic anemia at 9.2 g/dL with thrombocytopenia at 91 × 10^3^ elements/mm^3^ associated with inflammatory syndrome with C‐reactive protein level of 95 mg/L. Renal examination showed a renal failure with creatininemia at 37 mg/L and GFR of 17 mL/min/1.73 m^2^ (MDRDS) associated with a proteinuria at 0.51 g/24 h. Complementary analysis showed total protein level of 55 g/L with high alkaline phosphate and uric acid levels of 497 UI/L and 86 mg/L, respectively, and LDH at 221 UI/L. Dosing calcemia showed a hypercalcemia at 147 mg/L.

**Figure 1:**
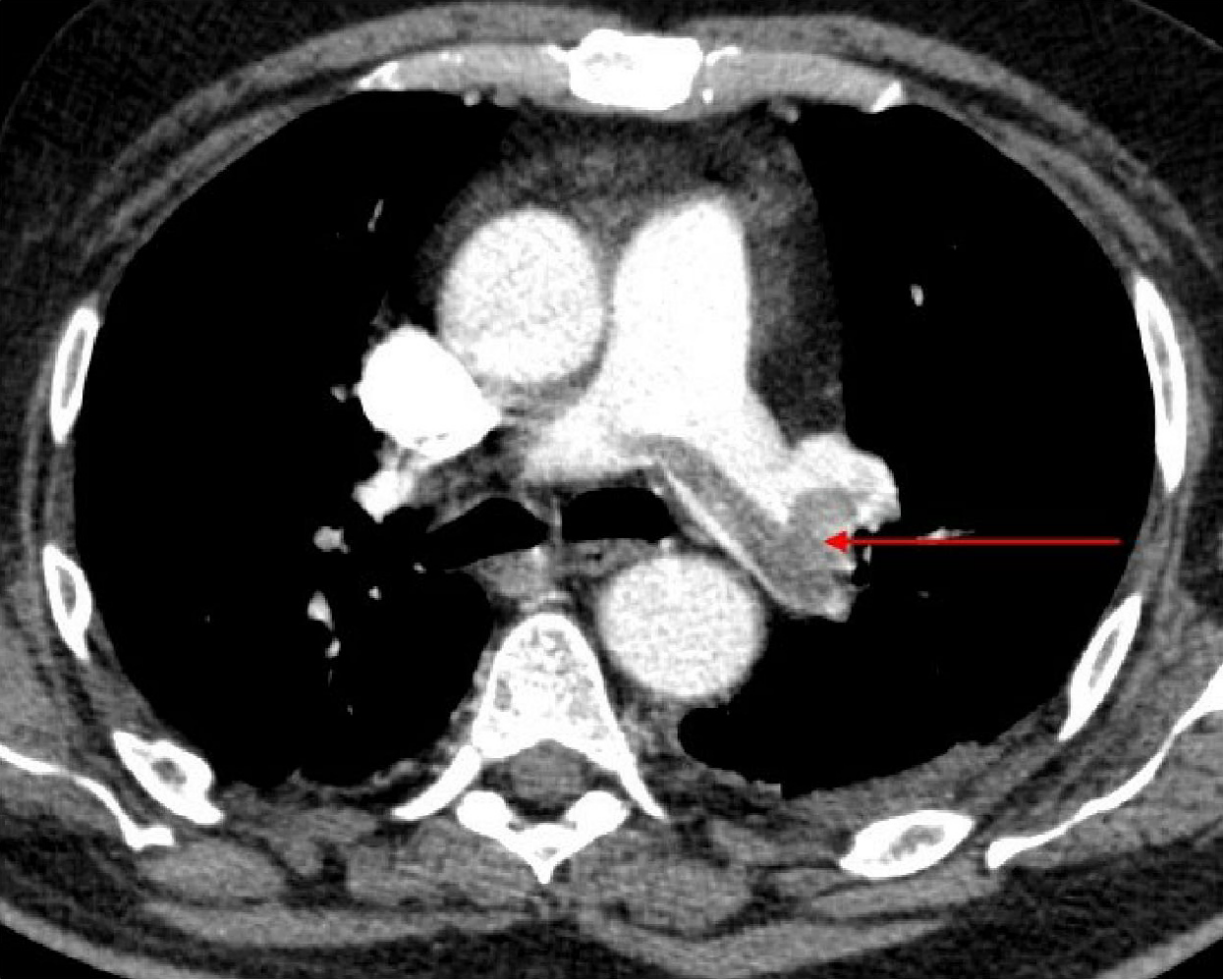
Computed tomography image showing pulmonary embolism.

Thoracic radiography on admission revealed the presence of bilateral basal foci that triggered the diagnosis of pulmonary embolism confirmed by thoraco‐abdominal angiography (Fig. 1). This later aided the accidental discovery of osteolytic lesions in the thoracic spine suspecting a multiple myeloma or bone metastasis.

**Figure 2:**
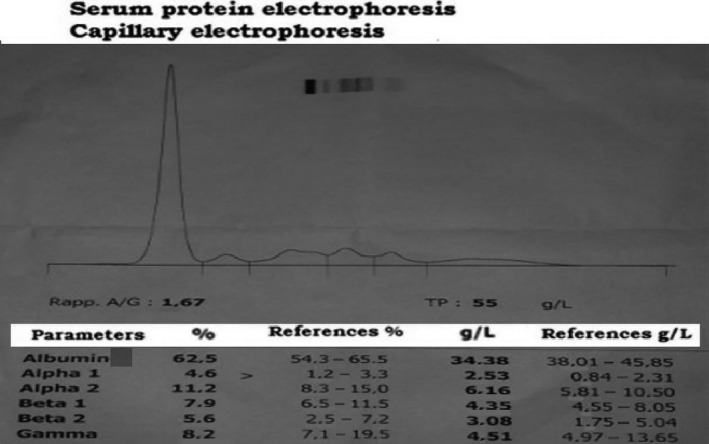
Serum protein electrophoresis showing hypogammaglobulinemia.

Gel electrophoresis of serum protein made on HYDRASYS of SEBIA^®^ gel showed the presence of hypogammaglobulinemia of 4.51 g/L, associated with a hypoalbuminemia at 33 g/L (Fig. 2).

**Figure 3:**
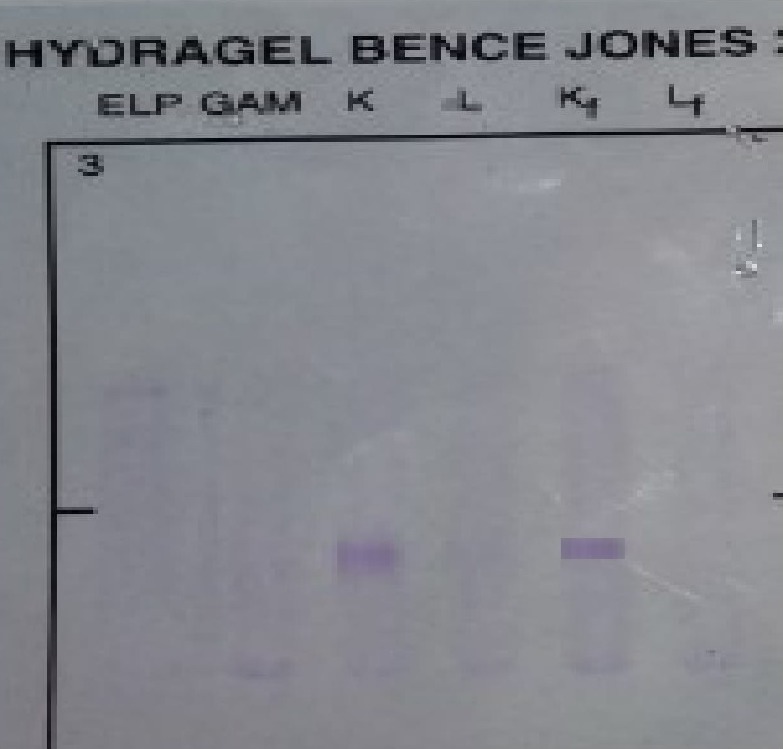
Urine immunofixation test showed the monoclonal‐free kappa light chains.

Immunofixation made on HYDRASYS of SEBIA^®^ gel showed no anomaly. Urine immunofixation made on HYDRASYS of SEBIA^®^ gel showed the presence of free monoclonal light chains of kappa isotype (Fig. 3).

Bone marrow examination revealed invasion of bone marrow by 12% of dystrophic plasma cells (on reserve of the marrow dilution). Kappa light‐chain myeloma was retained as the diagnosis.

The patient received emergency treatment with heparin and antibiotics while monitoring hypercalcemia.

While the patient's health was being enhanced, he was transferred to clinical hematology service for complementary care where he was put on velcade–thalidomide–dexamethasone as attack treatment.

## Discussion

Multiple myeloma is a malignant hemopathy in which a clonal population of plasma cells invades the bone marrow to more than 10%. Tumoral plasma cells can secrete intact monoclonal immunoglobulin or a light‐chain making, respectively, a multiple myeloma of intact immunoglobulin or of light chains 2, 3, 4.

Multiple myeloma is defined according to the recommendations of the International Myeloma Working Group, IMWG which appeared in 2014 by medulla plasmacytosis >10% (or a medullary or extramedullary plasmacytoma) and at least an event defining myeloma: at least a single CRAB criteria which are hypercalcemia, kidney failure, anemia, and bone lesions and or at least one marker of malignancy: focal lesions on MRI >1, bone marrow plasmacytosis ≥60%, a CLL ration involved (CLL i)/CLL not involved (CLL ni) ≥100.

The presence of urinary and or blood monoclonal protein is a nonobligatory criterion in the definition of myeloma; it helps to distinguish secretory myeloma from nonsecretory myeloma 3.

Our patient was admitted for acute respiratory distress. Given the context of operative follow‐up of a cholecystectomy 1 month ago, the diagnosis was oriented toward pulmonary embolism. The diagnosis of multiple myeloma is only occasionally posed by the discovery of osteolytic lesions in the cervical spine. Our patient had a bone marrow plasmacytosis rate of 12% and met all CRAB criteria with renal insufficiency at GFR of 17 mL/min/1.73 m2, hypercalcemia at 147 mg/L, and anemia at 9.2 g/dL. Thromboembolic venous disease is multifactorial. It has several risk factors such as age, obesity, long‐term hospitalization, or surgery. These factors in association with myeloma may interact with changes in hemostasis associated with this later in combination with hyperviscosity syndrome. During multiple myeloma, hyperviscosity is rarer, observed in 2–6% of cases, more frequently in the forms associated with IgA. The cases associated with IgE and light chains remain exceptional 5, 6. The thromboembolic risk is often due to the increased production of factor VIII, Willebrand factor, and pro‐inflammatory cytokines including CRP, interleukin 6, and interferon *α*. These cytokines alter the vascular endothelium and may therefore have procoagulant activity. 5 Regarding renal function, from a physiological point of view, the light chains are purified in 2–4 h by glomerular filtration. After filtration, they are either reabsorbed in intact form or metabolized into small peptides at the proximal convoluted tubule, which are then reabsorbed or eliminated in the urine.

In the case of saturation of the metabolism mechanism, the free light chains are found in the urine in the intact form 2.

Our patient was having a proteinuria at 0.51 g/24 h with the presence of kappa isotypes of monoclonal‐free light chains in the urinary immunofixation (Fig. 3). Serum electrophoresis does not show a monoclonal picture, but we notice the presence of hypogammaglobulinemia. The absence of a picture in the electrophoresis gives many explanations: Quantitatively serum electrophoresis only detects light chains from a concentration of 2000 mg/L, qualitatively, these light chains can polymerize, resulting in a sparse diffusion on the gel. In addition, the sensitivity of the electrophoresis to free light chains is very much dependent on the wideness of the electrophoretic band.

## Conclusion

Myeloma remains rare and incurable affection today. However, detailed understanding of the cellular mechanisms and the development of modern targeted therapies should enhance this worrying situation in the best near future.

Through this observation, we have wanted recall well that the combination of pulmonary embolism and myeloma remains possible in the case of hyperviscosity syndrome and that the pathological linkages between these two entities can be unusual and unverified within the already acquired information about patients.

## Authorship

BB, UJ, and EIS participated in manuscript concept and design, clinical assessment, acquisition of data, and drafting the manuscript. EA, DS, OE, and RA performed a literature review. AB, AD, BS, OZ, and DN revised the manuscript. All authors approved the final version of the manuscript.

## Conflict of Interest

None declared.
